# Association of Early Nutrition and Growth and Metabolic Parameters in Very Low Birth Weight Infants

**DOI:** 10.3390/nu18111719

**Published:** 2026-05-28

**Authors:** Indre Petraitiene, Rasa Brinkis, Egle Jurgaite, Ieva Sliauziene, Kastytis Smigelskas, Rasa Verkauskiene

**Affiliations:** 1 Institute of Endocrinology, Lithuanian University of Health Sciences, Medical Academy, LT-50161 Kaunas, Lithuania; 2 Department of Neonatology, Lithuanian University of Health Sciences, Medical Academy, LT-50161 Kaunas, Lithuania; 3 Health Research Institute, Faculty of Public Health, Lithuanian University of Health Sciences, LT-50161 Kaunas, Lithuania

**Keywords:** very low birth weight (VLBW), macronutrient, nutrition, very preterm (VP), extremely preterm (EP), growth, carbohydrate metabolism

## Abstract

Background/Objectives: Early nutrition is crucial for postnatal growth and metabolic status in preterm very low birth weight (VLBW) infants; however, the optimal macronutrient proportions remain unclear. We aimed to investigate how early nutrition influences long-term outcomes in extremely preterm (EP) and very/moderately preterm (VP) infants. Methods: We included 120 preterm infants in the prospective follow-up study. Anthropometric and metabolic parameters (fasting glycemia, insulin, and IGF-1) were assessed at birth, on the 28th day after birth, and once a year until 3–4 years (N = 65). Total daily parenteral and enteral nutrient intake was calculated. Standard deviation scores (SDS) for anthropometric measurements were calculated using Swedish growth reference data. Results: Although there was no difference in weight-adjusted macronutrient intake, EP newborns grew more slowly in the first 28 days than VP newborns. At the age of 1 year, the central-to-peripheral subcutaneous fat ratio was higher in children born EP compared to children born VP. No other anthropometric differences were found between groups at 1 year of age, and later between both groups. On the 28th day after birth, infants born EP had higher glucose and insulin levels, HOMA-IR, and lower IGF-1 levels compared to infants born VP. No relation was found between macronutrient intake and increases in weight SDS and height SDS in VP newborns. In the EP subgroup, carbohydrate and protein intake during the first 28 days were directly related to central-to-peripheral subcutaneous fat at 1 and 3–4 years. Conclusions: Early nutrition affects children’s growth up to 1 year of age, while later on, other factors seem to interfere. Higher protein and carbohydrate intake does not have a positive effect on the growth of preterm infants but is related to more central adipose tissue distribution.

## 1. Introduction

Approximately one in ten newborns is born prematurely, and very low birth weight (VLBW; <1500 g) infants present nearly 9% of all preterm births [1,2]. Advances in perinatal care have improved the survival rate in this population [3,4]. However, VLBW infants remain at high risk for multiple complications, including infection, intracranial hemorrhage, necrotizing enterocolitis, bronchopulmonary dysplasia, cystic periventricular leukomalacia, retinopathy, and impaired growth [3,5]. These infants are delivered during a critical window of fetal growth when the placenta is the primary source of nutrients. The abrupt transition from placental to postnatal nutrition, combined with concurrent morbidities, frequently results in postnatal growth restriction and failure to maintain birth growth percentile [5,6,7]. Longitudinal studies indicate that growth deficits may persist into childhood [8].

Insulin-like growth factor-1 (IGF-1) is central to postnatal linear growth in preterm infants. Persistently low IGF-1 levels, relative to intrauterine norms, have been associated with poor growth outcomes in VLBW infants [9,10,11], including a reduced likelihood of attaining target percentiles by school age [8]. These mechanistic insights highlight that hormonal dysregulation, particularly of IGF-1, may underpin the link between preterm birth, disrupted nutritional supply, and impaired longitudinal growth.

Beyond growth, preterm and VLBW infants exhibit early signs of metabolic dysregulation. From school age onwards, these individuals have higher rates of insulin resistance and increased blood pressure compared with term-born peers [12], as well as at an increased risk of developing both type 1 and type 2 diabetes in adulthood [13]. Several studies further report increased fasting glucose, insulin, and HOMA-IR (homeostatic model assessment for insulin resistance) in adults born preterm [14,15], though findings are not entirely consistent [16]. The etiology of these long-term metabolic sequelae remains incompletely understood, and evidence for specific perinatal determinants is conflicting.

Early nutrition is a critical, modifiable factor potentially influencing both growth and metabolic outcomes in VLBW infants. The primary clinical objective is to match intrauterine growth rates and prevent postnatal growth restriction and long-term metabolic complications [17]. However, meeting the high and specific nutrient requirements of VLBW infants remains challenging, and uncertainties persist regarding optimal macronutrient composition.

Parenteral nutrition is often required immediately after birth, yet enteral feeding—ideally with maternal milk—is preferred when feasible. Because maternal milk alone may not fulfill the elevated requirements of VLBW infants, targeted fortification is routinely employed [18]. Evidence suggests that higher early protein intake can improve weight gain up to term-equivalent age, though data on other macronutrients and on longer-term impacts are inconsistent [7,19,20]. Some studies report enhanced lean mass with early protein and energy intakes [21], but results are not universal. Higher fat intake may transiently increase fat mass [22], but the long-term metabolic consequences remain unclear. Moreover, there is limited and inconsistent evidence that early nutrition—particularly higher carbohydrate intake—influences later growth or metabolic parameters such as insulin sensitivity [23,24,25].

Consequently, despite the recognized importance of early, balanced nutrition in VLBW infants, knowledge gaps remain. Specifically, the optimal amounts and proportions of macronutrients for promoting healthy growth without adversely affecting future metabolic health are not established. There is a paucity of data on the effects of macronutrient composition—not just total energy or protein intake—on the longitudinal trajectories of growth and metabolism. In addition, it is unclear whether optimal nutritional strategies should be adjusted for gestational age subgroups such as extremely preterm (EP, <28 weeks), very preterm (28–31 weeks), or moderately preterm (32–34 weeks) infants.

The present study aims to address these gaps by assessing nutrient intake during the first 28 days of life in VLBW infants and evaluating associations with growth and metabolic outcomes up to 3–4 years of age. We hypothesized that early macronutrient intake would influence both intermediary and longer-term growth and metabolic status in this vulnerable population.

## 2. Materials and Methods

### 2.1. Study Design and Population

This prospective, non-interventional observational study was conducted at the Neonatology Department of Kaunas Clinics, Lithuanian University of Health Sciences Hospital. This study included infants born between 31 May 2018 and 17 May 2020. Inclusion criteria were birth weight < 1500 g, gestational age ≤ 34 weeks, and the written consent of both parents. Exclusion criteria were chromosomal abnormalities, genetic syndromes affecting growth, surgery with partial bowel removal, and the absence of written consent from both parents [7].

Among 120 enrolled infants, 8 died during the neonatal period, and 3 underwent partial bowel resection due to necrotizing enterocolitis, resulting in 109 infants eligible for follow-up. The cohort was followed up until 4 years of age. Study subjects were assessed at 28 days after birth and at 1, 2, 3, and 4 years of age. Due to the small numbers of subjects at 3 and 4 years, data from these follow-up visits were combined into a single group for analysis (Table 1).

For statistical analyses, study subjects were categorized into two subgroups based on gestational age: extremely preterm (EP, <28 weeks) and very/moderately preterm (VP, 28–34 weeks). The incidence of early complications related to prematurity differed between the groups. Early and late sepsis, respiratory distress syndrome, bronchopulmonary dysplasia, and patent ductus arteriosus were more frequently detected in newborns in the EP group (26.4% vs. 7.5%, *p* = 0.005; 28.3% vs. 6%, *p* < 0.001; 83% vs. 35.8%, *p* < 0.001; 11.3% vs. 1.5%, *p* = 0.021; 60.4% vs. 13.4%, *p* < 0.001, respectively).

### 2.2. Feeding Practices and Nutritional Calculations

Parenteral nutrition was started as soon as venous access was obtained. Enteral nutrition with mother’s milk or donor milk was started within the first hours of life. Fresh colostrum (0.2 mL) was applied orally before feedings. The starting volume of milk was 20 mL/kg/day, with daily advancements of 20–30 mL/kg. With advancing enteral feeds, parenteral nutrition was gradually weaned. Standard human milk fortification with a bovine-based powdered human milk fortifier (Aptamil FMS^®^, Milupa/Danone GmbH, Friedrichsdorf, Germany, Danone Nutricia, Cuijk, The Netherlands) was introduced after full enteral feeding was achieved (at a median time of 7 days) and continued until hospital discharge. Analysis of the mother’s milk was carried out twice a week using a mid-infrared spectroscopy human milk analyzer (MIRIS, Uppsala, Sweden). All donor milk samples were analyzed for nutrients. Total daily nutrient intake consisted of parenteral and enteral intakes calculated from actual data in medical records. All infants were fed following the standard clinical practice guidelines [7].

### 2.3. Growth Assessments

Anthropometric measurements were performed at birth, on the 28th day after birth, and at 1, 2, 3, and 4 years of age, with corrected age applied for the assessments at 1 and 2 years.

During the first year, infants were weighed using electronic infant scales (Marsden, Rotherham, UK), and length was measured in the supine position with an infant measuring rod (SECA, Hamburg, Germany). From 2 years of age, weight was measured using standard electronic standing scales (Charder, Taigung City, Taiwan), and standing height was assessed with a Harpenden stadiometer (Holtain Limited, Crymych, UK). Body mass index (BMI, kg/m^2^) was calculated at each time point. Waist circumference was measured at 3 and 4 years of age with a measuring tape (SECA, Hamburg, Germany).

Skinfold thickness was measured at four sites (biceps, triceps, subscapular, and suprailiac) on the right side of the body using a calibrated Harpenden calliper (Baty International, Burgess Hill, UK) with a constant pressure of 10 g/mm^2^; the mean of two measurements was used for analysis.

The central-to-peripheral subcutaneous fat ratio was calculated as the sum of subscapular and suprailiac skinfolds (central) divided by the sum of biceps and triceps skinfolds (peripheral).

Target height (TH), or mid-parental height, was calculated according to the standard formula, adjusted for the child’s sex (father’s height + mother’s height + 13 cm for boys or −13 cm for girls, divided by 2).

Standard deviation scores (SDS) for length, height, weight, and target height were calculated using Swedish growth reference data adjusted for age and sex [26,27].

### 2.4. Hormonal and Metabolic Analysis

Laboratory assessments of glycemia, insulin, and IGF 1 were performed at all study time points except at birth. In infants, blood samples were collected on day 28 between 08:00 and 12:00 p.m., before the feeding. In later childhood, venous blood was drawn after an overnight fast at the same morning hours. All hormone samples were centrifuged within 1 h and stored at −20 °C until analysis. Plasma glucose samples were delivered immediately to the laboratory and analyzed within 2 h using the oxygen coefficient method on a Synchron biochemical analyzer (Beckman Coulter, Brea, CA, USA). Insulin concentrations were measured using an immunoradiometric assay (IRMA DIAsource, Belgium; detection limit 1.0 mIU/L; intra-assay CV 1.9%; inter-assay CV 6.3%), and IGF-1 concentrations were measured using a radioimmunoassay (RIA DIAsource, Belgium; detection limit 3.4 µg/L; intra-assay CV 4.3%; inter-assay CV 6.5%) in a certified clinical laboratory. Insulin resistance was estimated using the homeostatic model assessment for insulin resistance (HOMA-IR), calculated as [fasting plasma glucose (mmol/L) × fasting plasma insulin (mU/L)]/22.5 [28].

### 2.5. Statistical Analysis

Statistical analyses were performed using Microsoft Excel version 16.81 and IBM SPSS Statistics for Windows (version 29.0.1.0, IBM Corp., Armonk, NY, USA). Descriptive analysis for normally distributed variables included means and standard deviations (SDs), medians and interquartile ranges (IQRs) for those without normal distribution, and percentages for categorical indicators. The normality of variable distribution was estimated using skewness and kurtosis. For group comparisons, the parametric Student’s *t*-test for normally distributed values and the non-parametric Mann–Whitney U test for values without normal distribution were employed. For comparison of categorical indicators, the Chi-squared test was used. The missing data were handled pairwise to improve power. The relationships between two continuous variables were assessed using Spearman’s correlation coefficient. The statistical significance was set at *p* < 0.05.

## 3. Results

*Study population.* We examined 57 boys and 63 girls (47.5%/52.5%). Children were born at 22–34 weeks of gestation (mean: 27.8 ± 2.5 weeks). From the entire cohort, 53 subjects (43.8%) were born extremely preterm (EP) and 67 subjects (55.8%) were born very/moderately preterm (VP). There was no difference in the proportion of boys and girls between the EP (55% and 45%) and the VP groups (42% and 58%, *p* = 0.159). The mean gestational age of those born EP was 25.5 ± 1.4 weeks, and 29.6 ± 1.6 weeks for those born VP. There was no difference in macronutrient intake between EP and VP groups (Table 2).

**Figure 1:**
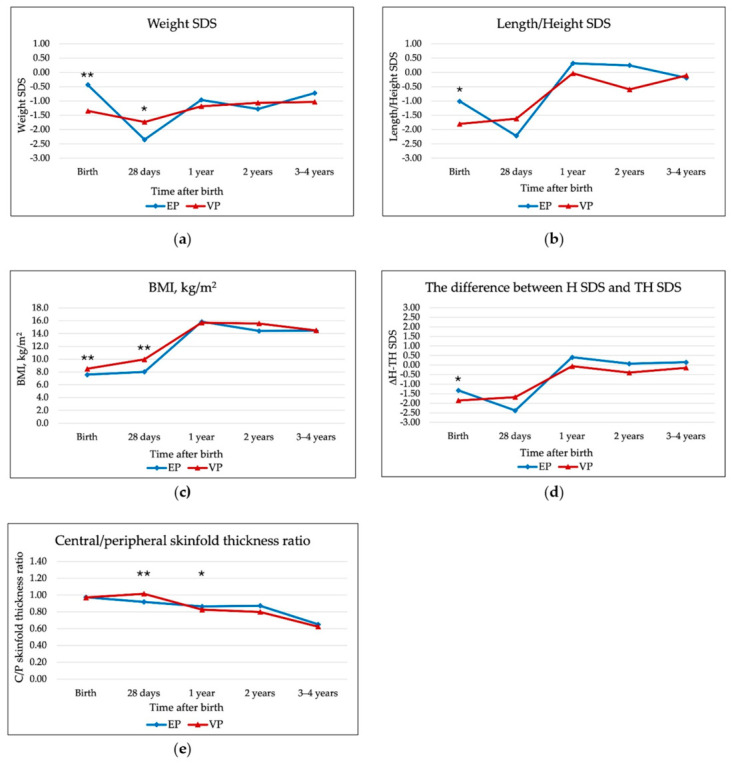
Change in weight SDS (**a**), length/height SDS (**b**), body mass index (BMI) (**c**), the lag of height SDS from the target height SDS (∆H-TH SDS) (**d**), and the ratio between central-to-peripheral skinfold thickness (**e**) in children born extremely preterm (EP) and very/moderately preterm (VP) (* *p* < 0.05; ** *p* < 0.001).

*Anthropometric parameters.* At birth and at the 28th day after birth, infants born EP were lighter and shorter than infants born VP (birth weight: 0.87 ± 0.21 vs. 1.24 ± 0.16 kg, *p* < 0.001; birth length: 33.70 ± 2.69 vs. 38.05 ± 1.96 cm, *p* < 0.001, respectively), but their weight SDS and length SDS were significantly higher compared to children born VP. The lag of height SDS from the target height SDS (∆H-TH SDS) was also smaller in newborns born EP compared to newborns born VP (Figure 1). At the 28th day after birth, EP children remained lighter and shorter than children born VP (weight 1.21 ± 0.26 vs. 1.81 ± 0.21 kg, *p* < 0.001, and length 38.43 ± 2.93 vs. 42.77 ± 1.52 cm, *p* < 0.001, respectively). The difference in weight SDS at the 28th day after birth remained, but the difference in length SDS did not reach statistical significance (Figure 1). During the first 28 days of life, there was a significant decrease in weight SDS, height SDS, and ∆H-TH SDS (all *p* < 0.001) in the EP group, whereas in the VP group, there was a decrease in weight SDS (*p* = 0.023) but no significant differences in height SDS and ∆H-TH SDS. EP children had a lower BMI at birth and on the 28th day after birth (Figure 1). Later, there were no differences in weight SDS, height SDS, ∆H-TH SDS, and BMI levels between EP and VP groups (Figure 1).

At birth, there was no difference in the central-to-peripheral subcutaneous fat ratio between EP and VP newborns. At the 28th day after birth, children born EP had a lower central-to-peripheral subcutaneous fat ratio compared to children born VP. At 1 year of age, the central-to-peripheral subcutaneous fat ratio was higher in children born EP than in children born VP, but the difference disappeared in subsequent measurements (Figure 1). There was no difference in waist-to-height ratio in children born EP and VP at the age of 3–4 years (0.49 [0.48–0.51] vs. 0.48 [0.46–0.50], *p* = 0.068).

*Metabolic parameters.* Children born EP had lower IGF-1 levels at the 28th day after birth compared to children born VP (Table 3). Later, there was no difference in IGF-1 levels between the two groups. Comparing parameters of glucose metabolism between EP and VP groups, infants born EP had higher glucose and insulin levels and HOMA-IR at 4 weeks after birth compared to infants born VP (Table 2). Only one child had glycemia higher than 10 mmol/L at the 28th day after birth (12.7 mmol/L, from the EP group). Still, even after removing that child’s data from the analysis, the difference between the groups remained significant. There was no difference in metabolic parameters at 1, 2, and 3 to 4 years of age (Table 3).

*Relation to early nutrition.* In the VP subgroup, macronutrient intake during the first 28 days of life was not associated with anthropometric measurements (Table 4). At 1 year of age in EP children, weight SDS and BMI were related to the total amount of carbohydrate and fat, but not to the total amount of protein. In addition, fat intake during the first 28 days of life was directly related to height SDS at 1 year of age and weight SDS and BMI at 2 years of age. In the EP subgroup, carbohydrate and protein intake during the first 28 days of life were directly related to central-to-peripheral subcutaneous fat at 1 and 3–4 years. There was a positive association of the total amount of every macronutrient during the first 28 days of life with ∆H-TH SDS at 1 year of age in children born EP. Such a relationship was also found in 3–4-year-old EP children (Table 4).

At the 28th day after birth, the total amount of protein during the first 28 days after birth was directly related to BMI and IGF-1 levels in children born EP, but not in children born VP (Table 4). In addition, in EP children, fat intake was also directly associated with IGF-1 levels.

The negative association of protein intake with glycemia was evident in the VP subgroup at the 28th day after birth (Table 4). In EP children, there was an inverse association of carbohydrate and fat intake with fasting insulin and HOMA-IR at that age.

In the EP subgroup, there was no association between macronutrient intake and fasting glycemia from 1 year of age onwards or insulin levels at 3–4 years of age. There was an inverse correlation between carbohydrate and protein intake and fasting glycemia at 2 years of age in the VP group (Table 4).

*Relations to size at birth.* Weight SDS and height SDS at 3–4 years of age were directly related to weight SDS and height SDS at birth (weight SDS at 3–4 years: rho = 0.350 (95% CI: 0.116–0.549), *p* = 0.005 and rho = 0.255 (95% CI: 0.019–0.467), *p* = 0.042, respectively; height SDS at 3–4 years: rho = 0.402 (95% CI: 0.192–0.569), *p* = 0.001 and r = 0.366 (95% CI: 0.154–0.539), *p* = 0.003, respectively). In addition, in children born VP, weight SDS and height SDS at 3–4 years of age were inversely related to gestational age (rho = −0.388 (95% CI: −0.638–(−0.092)), *p* = 0.011, and rho = −0.491 (95% CI: −0.694–(−0.210)), *p* < 0.001, respectively). In the EP subgroup, the relation of height SDS at 3–4 years and height SDS at birth remained, but the association between current height SDS and birth weight did not reach statistical significance. There was no association between weight SDS at 3–4 years and size at birth in the EP group.

At 3–4 years of age, BMI, central-to-peripheral subcutaneous fat, waist-to-height ratio, glycemia, insulin, HOMA-IR, and IGF-1 levels were not related to gestational age or size at birth in the total cohort and in subgroup analysis.

## 4. Discussion

The main finding of this study is that despite there being no difference in macronutrient intake, EP newborns grew more slowly in the first 28 days than VP newborns. However, there was no difference in height SDS, weight SDS, and BMI after 1 year of age between the two groups. The higher SDS for length and weight at birth in EP infants could be explained by a shorter period of adverse conditions in the womb. However, the rapid loss of birth weight SDS, BMI, and the slowing of height growth after birth reflect the high stress experienced due to extreme prematurity and a higher number of medical interventions. Meanwhile, VP children did not show a significant decrease in weight during the first 28 days after birth, and the rise of height SDS was already present during the first 28 days of life. From the age of 1 year, in children from both groups, the increase in height SDS and weight SDS stabilized.

According to most current recommendations for the early enteral nutrition of premature newborns, protein intake is of particular importance. Protein intake is considered to be the main driver of lean body mass growth. The study by Fu showed that VLBW infants would likely benefit from more aggressive and earlier fortification strategies that target protein supplementation, as infants fed calorically targeted donor breast milk demonstrate a disproportionate negative change in length z-score over time [29]. Other studies in newborns have shown that a higher protein intake is associated with better weight gain [30,31,32,33]. However, the data on the impact of protein on height growth are not consistent. According to the observations of Cormack, high enteral protein intake promotes weight and length gain, but the association between length gain and protein intake was reversed if the protein was administered parenterally [19]. In contrast to these studies, we found no relation between protein intake and an increase in weight SDS and height SDS at the 28th day after birth and onwards in subgroups. Although there was a positive association between protein intake and serum IGF-1 levels at the 28th day after birth in EP children, it was also linked to a greater increase in BMI until the 28th day after birth and to a higher central-to-peripheral fat ratio at 1 and 3–4 years of age. Thus, higher protein supplementation during the neonatal period for EP children could also have disadvantages.

The greater weight gain relative to length in premature infants and its relation to overweight, obesity, and subsequent metabolic disorders later in life have also been emphasized by some authors [32,34]. Faster weight gain in premature newborns is achieved by feeding larger amounts than the recommended human milk-based strategy [34]. The adipogenic effect of supplemented protein is associated with an increase in the levels of insulin-releasing amino acids, which in turn stimulate insulin and IGF-1 secretion [32]. Studies have shown that high protein intake at 1 year of age is associated with higher body fat mass at 6 years of age [35]. This association remained significant even after adjustment for BMI at the age of 1 year and was stronger among children who had catch-up growth in the first year of life [35]. The association between an increase in energy from protein and a higher waist-to-hip ratio was also noted by other authors in older children [36]. We found neither a positive nor a negative association between higher protein intake and growth or metabolic parameters in VP children. Since higher protein intake is associated with faster weight gain, we conclude that high amounts of protein supplementation may not increase benefits in newborns born VP.

It might be hypothesized that in EP children, a decrease in central-to-peripheral subcutaneous fat in the first 28 days of life and a subsequent increase until 1 year may be related to the prolonged period of excessive stress. Later on, the ratio of central and peripheral fat equalizes in both groups. In addition, there was no association between central-to-peripheral subcutaneous fat ratio and gestational age, suggesting that fat mass distribution is more influenced by factors other than the duration of gestation. Feeding regimens focusing on rapid weight gain may result in both a transient high proportion of body fat and possibly an early-childhood adiposity rebound [34,37,38]. In our study, we did not find any relation between carbohydrate intake and body composition characteristics at any evaluated age in children born VP. However, in EP children, carbohydrate intake had a direct relationship with weight SDS, BMI, and central-to-peripheral subcutaneous fat ratio at 1 year of age, and with unfavorable subcutaneous fat distribution, but no other parameters of worse metabolic status, at 3–4 years of age. This suggests that characteristics of body composition from the age of 2 years are more influenced by factors other than early nutrition. However, the relationship between carbohydrate intake and central-to-peripheral subcutaneous fat ratio warns of the potential negative impact of a higher carbohydrate intake on later unfavorable body composition in EP children and needs further investigation in older children.

Higher postnatal stress in the EP group is also indicated by higher glycemia, insulin, and HOMA-IR on day 28th after birth. High stress increases insulin resistance and glycemia. After a period requiring many medical interventions, the indicators of carbohydrate metabolism became equal in both groups of children.

The study by Tottman showed that in very preterm infants, reduced fat, carbohydrate, and energy intake, as well as higher protein intake and small, early enteral feeds, may assist glycemic control [39]. However, in our study, carbohydrate and protein intake were inversely related to glycemia on the 28th day after birth. This may be explained by the fact that newborns whose glycemia dropped received more nutrients. We did not analyze the incidence of hypoglycemia in infancy, but at the same time, we did not find any relationship between protein intake and insulin and HOMA-IR on the 28th day after birth. Furthermore, it was not protein intake but carbohydrate intake that was inversely related to HOMA-IR in infants born EP. We cannot conclude that carbohydrate intake is protective against insulin resistance; however, we also did not find signs that protein-enriched nutrition had a positive effect on carbohydrate metabolism on the 28th day after birth and until 3–4 years of age. Since metabolic disorders may occur in older age, longer follow-up studies are needed to assess whether early nutrition could have an impact on long-term metabolic outcomes. We cannot explain the inverse relationship between carbohydrate and protein intake during the first 28 days and glycemia at 2 years of age in the VP group. Since we did not observe similar trends at 1 year of age or at 3–4 years of age, this may represent an inaccuracy due to the small number of subjects.

The mother’s own milk has the ideal nutritional composition for the infant, and multi-nutrient fortifiers may alter this balance. All infants in our study received standard fortification. The macronutrients in the fortifier were protein and carbohydrates. The fat was provided solely by human milk. Our study findings suggest that each macronutrient may have a different effect on metabolic markers later in life; however, only minor associations were observed in very/moderately preterm infants. Further studies should evaluate the necessity of fortification in the specific groups of preterm infants.

### Strengths and Limitations

The strengths of this study are that the children were followed prospectively from birth to 3–4 years of age and that the accurate calculation of macronutrients in human milk and supplemental food was performed for all newborns up to 28 days of life.

The main limitation of this study was a considerable drop-out from the follow-up, resulting in only 65 cohort subjects examined between the ages of 3 and 4 years. Another limitation of this study was that when examining children aged one and two, we could not ensure that the tests were truly taken after fasting for at least 4 h. Therefore, we did not compare the blood levels of insulin and IGF-1 in children of this age. In addition, over time, there are more factors that influence a child’s growth, such as socioeconomic status, nutrition, and physical activity. Since the number of subjects was small, we did not include controlling factors in the calculation in order not to reduce the statistical power. Furthermore, the mother’s milk was used for early enteral feeding to maximize the benefits of colostrum and was not analyzed for nutritional composition until the end of the first week. Therefore, the total nutrient intake of the first week is underestimated.

## 5. Conclusions

Early nutrition appears to be associated with growth in children up to 1 year of age, while growth beyond infancy is likely influenced by additional factors. Suboptimal growth in extremely preterm infants may reflect not only nutritional challenges but also the impact of increased medical interventions and associated stress. Differences in the metabolic profiles of extremely preterm versus very preterm infants were observed at 28 days of age, but these differences were not evident at later follow-up.

In this cohort, higher dietary protein levels were not consistently linked to improved growth outcomes in preterm infants. However, the precise effects of protein supplementation or fortification remain uncertain, particularly given limitations in data regarding the specific amounts of nutrients provided by fortifiers. Additional studies capturing accurate fortifier composition and administered amounts would help clarify the individualized nutrient requirements of VLBW infants and inform evidence-based nutritional strategies.

## Figures and Tables

**Table 1:** Number of studied infants and children in all age groups.

Age	At Birth	28th Day	1 Year	2 Years	3–4 Years
N	120	102	95	53	65

**Table 2:** Macronutrient intake during the first 28 days in infants born extremely preterm (EP) and very/moderately preterm (VP). Data are presented as the mean ± SD.

Macronutrient Intake	EP (N = 52)	VP (N = 67)	*p*
Cumulative carbohydrate intake (g/kg)	322.7 ± 102.5	339.3 ± 70.1	0.319
Cumulative protein intake (g/kg)	79.2 ± 23.7	82.0 ± 18.3	0.467
Cumulative fat intake (g/kg)	127.9 ± 47.1	131.3 ± 34.6	0.652

**Table 3:** Parameters of glucose metabolism and insulin-like growth factor-1 (IGF-1) levels in children born extremely preterm (EP) and very/moderately (VP) preterm. Data are presented as the median and interquartile range. (HOMA-IR—homeostatic model assessment for insulin resistance).

Parameters	EP	VP	*p*
**28th day after birth**			
Fasting glycemia (mmol/L)	4.70 [4.05–5.65]	4.30 [3.50–5.15]	**0.008**
Fasting insulin (mU/L)	12.77 [10.33–19.90]	11.22 [7.41–16.98]	**0.038**
HOMA-IR	2.90 [2.09–5.62]	1.89 [1.06–3.71]	**0.017**
IGF-1 (nmol/L)	1.04 [0.49–1.69]	1.98 [1.42–2.89]	**<0.001**
**1 year**			
Fasting glycemia (mmol/L)	5.00 [4.70–5.40]	5.00 [4.60–5.20]	0.537
IGF-1 (nmol/L)	5.83 [3.86–8.35]	5.60 [3.63–7.16]	0.451
**2 years**			
Fasting glycemia (mmol/L)	4.90 [4.60–5.25]	4.80 [4.70–5.30]	0.923
**3–4 years**			
Fasting glycemia (mmol/L)	4.47 [4.13–4.12]	4.37 [3.91–4.65]	0.424
Fasting insulin (mU/L)	1.95 [1.50–3.00]	2.20 [1.20–3.20]	0.863
HOMA-IR	2.90 [2.09–5.62]	1.89 [1.06–3.71]	0.954
IGF-1 (nmol/L)	7.29 [3.12–10.68]	7.63 [4.53–12.60]	0.337

Bold text indicates the age at assessment and statistically significant differences between groups.

**Table 4:** Relationship of early nutrition during the first 28 days with anthropometric and metabolic parameters at 3–4 years of age. Data are presented as Spearman’s rho. Significant correlations are highlighted. (BMI—body mass index, HOMA-IR—homeostatic model assessment for insulin resistance, IGF-1—insulin-like growth factor-1, ∆H-TH SDS—the lag of height SDS from the target height SDS) (* *p* < 0.05; ** *p* < 0.01).

	Extremely Preterm			Very/Moderately Preterm		
Parameters	Carbohydrate	Fat	Protein	Carbohydrate	Fat	Protein
**Day 28**						
Height SDS	0.22	0.18	0.00	0.09	−0.02	−0.14
Weight SDS	0.15	0.14	0.08	0.22	0.04	0.04
BMI	0.29	0.19	0.31 *	0.18	0.06	0.19
Central-to-peripheral subcutaneous fat	−0.04	−0.23	−0.10	−0.07	0.13	−0.06
Fasting glycemia	−0.21	−0.27	−0.07	−0.22	−0.10	−0.33 *
HOMA-IR	−0.32 *	−0.39 *	−0.13	−0.09	−0.12	−0.02
IGF-1	0.23	0.34 *	0.35 *	0.10	0.18	0.08
∆H-TH SDS	0.29	0.19	0.11	0.18	0.13	0.01
**1 year**						
Height SDS	0.29	0.41 **	0.16	0.02	0.04	0.06
Weight SDS	0.46 **	0.42 **	0.30	−0.14	−0.04	−0.15
BMI	0.45 **	0.39 *	0.30	−0.13	−0.04	−0.13
Central-to-peripheral subcutaneous fat	0.43 **	0.13	0.34 *	−0.26	−0.04	−0.21
Fasting glycemia	−0.23	−0.20	0.07	0.05	0.04	0.10
IGF-1	0.16	−0.14	0.06	−0.23	−0.16	−0.26
∆H-TH SDS	0.49 **	0.48 **	0.43 **	0.02	0.15	0.12
**2 years**						
Height SDS	0.34	0.24	0.14	−0.08	−0.13	−0.14
Weight SDS	0.27	0.54 **	0.29	−0.16	−0.20	−0.06
BMI	0.15	0.46 *	0.29	−0.04	−0.01	0.17
Central-to-peripheral subcutaneous fat	0.32	0.35	0.17	−0.33	−0.25	−0.26
Fasting glycemia	0.02	0.16	−0.02	−0.40 *	−0.34	−0.51 **
∆H-TH SDS	0.47 *	0.30	0.42	−0.14	0.02	−0.10
**3–4 years**						
Height SDS	0.30	0.41	0.26	−0.13	−0.11	−0.12
Weight SDS	0.26	0.29	0.14	−0.20	−0.21	−0.24
BMI	0.08	0.26	0.10	−0.14	−0.09	−0.21
Central-to-peripheral subcutaneous fat	0.46 *	−0.19	0.44 *	−0.08	−0.04	−0.14
Waist to height ratio	−0.02	0.14	0.11	−0.06	0.04	−0.18
Fasting glycemia	−0.12	−0.12	−0.06	−0.01	0.05	−0.08
HOMA-IR	0.29	−0.09	0.14	−0.17	0.00	−0.19
IGF-1	0.44	−0.18	0.26	−0.10	0.00	0.10
∆H-TH SDS	0.60 **	0.44 *	0.50 *	0.15	0.02	0.01

Bold text indicates the age at assessment and statistically significant differences between groups.

## Data Availability

The database is held by the authors and is available upon reasonable request for a justified purpose.

## Abbreviations

The following abbreviations are used in this manuscript:

VLBW	low birth weight
EP	extremely preterm
VP	very/moderately preterm
SDS	standard deviation scores
BMI	body mass index
TH	target height
∆H-TH SDS	the lag of height SDS from the target height SDS
HOMA-IR	homeostatic model assessment for insulin resistance
IGF-1	insulin-like growth factor-1
